# miR-215 functions as a tumor suppressor and directly targets ZEB2 in human non-small cell lung cancer

**DOI:** 10.3892/ol.2021.12861

**Published:** 2021-06-10

**Authors:** Yan Hou, Junwen Zhen, Xiaodong Xu, Kun Zhen, Bin Zhu, Rui Pan, Chidong Zhao

Oncol Lett 10: 1985-1982, 2015; DOI: 10.3892/ol.2015.3587

Following the publication of this paper, it was drawn to the Editors’ attention by a concerned reader that the Transwell migration assay data shown in Fig. 2D, and the scratch wound assay data shown in in Fig. 2E, were strikingly similar to data appearing in different form in other articles by different authors at different research institutes. Owing to the fact that the contentious data in the above article were already under consideration for publication elsewhere prior to its submission to *Oncology Letters*, the Editor has decided that this paper should be retracted from the Journal. The authors were asked for an explanation to account for these concerns, but the Editorial Office never received any reply. The Editor apologizes to the readership for any inconvenience caused.

